# Supplementary material for: Perturbed Wnt signaling leads to neuronal migration delay, altered interhemispheric connections and impaired social behavior
Source: Nat Commun. 2017 Oct 27;8:1158. doi: 10.1038/s41467-017-01046-w (PMC5660087; doi:10.1038/s41467-017-01046-w)
Supplement: Supplementary file 1 — Supplementary Information [file 41467_2017_1046_MOESM1_ESM.pdf]

Supplementary Figure 1

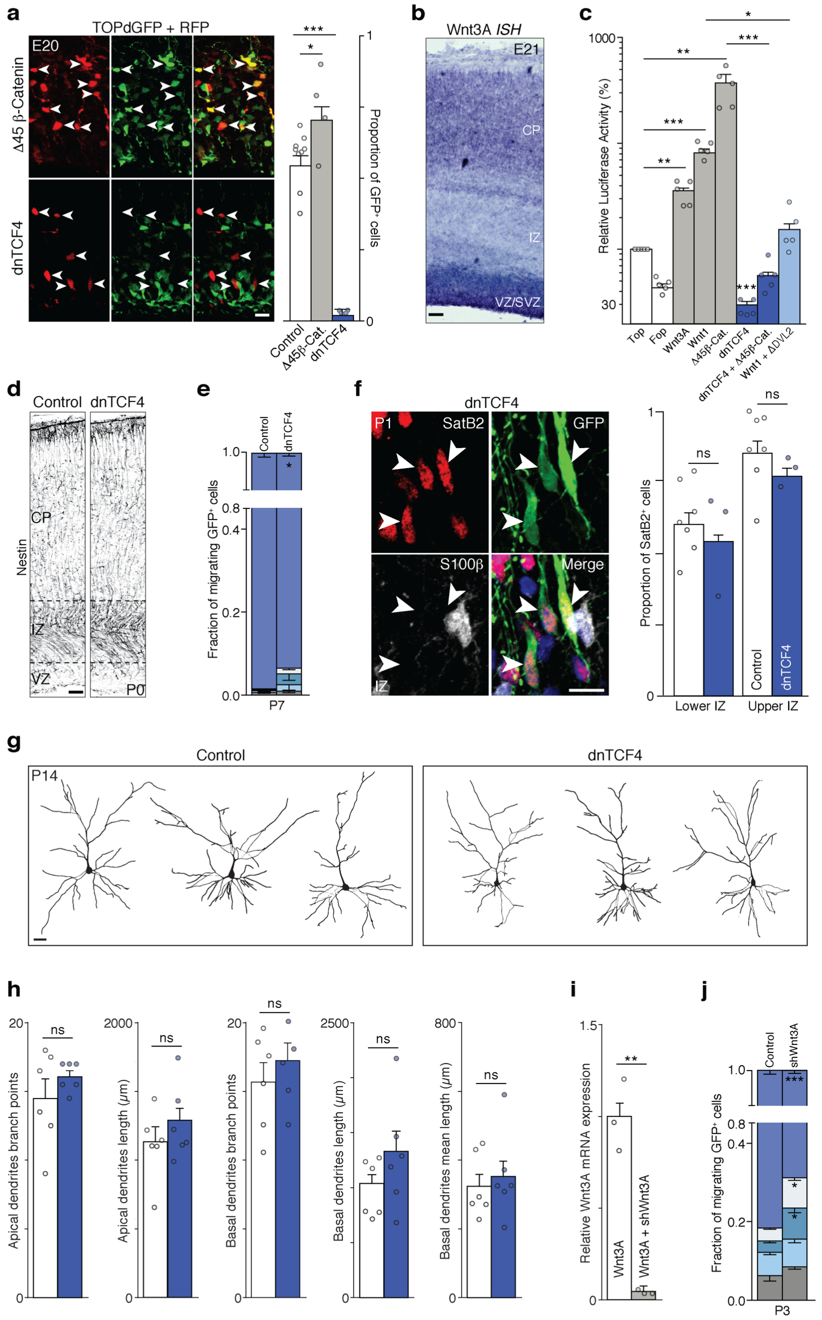

Characterization of cells with Wnt loss-of-function and validation of plasmids. (a) E20 in vivo confocal images of co-electroporated neurons with TOPdGFP and dnTCF4 or Δ45β-catenin plasmids. Graphic represents the proportion of GFP/RFP positive cells in control, Δ45β-catenin and dnTCF4 conditions; n = 9 brains for control (2312 cells), 4 for Δ45β-catenin (619 cells) and 3 for dnTCF4 (464 cells) from 2 experiments, 1way ANOVA. **(b)** In situ hybridization on wild-type E21 brains for Wnt3A. **(c)** In vitro validation of plasmids by Luciferase Top Flash Assay. TOP condition reflects endogenous signaling levels in 293T-transfected cells; whereas FOP mutated promoter condition represent the basal non-specific transcriptional activation; n = 5 experiments, paired t-test. **(d)** P0 confocal images of Nestin staining following E18 electroporation illustrating radial fiber integrity. **(e)** Analysis of cell distribution at P7 (see also Fig. 1e); n = 9 and 7 brains (Control, dnTCF4, respectively), 2way ANOVA. **(f)** P1 confocal images of brain sections showing dnTCF4 electroporated neurons (GFP, arrowheads) with positive SatB2 and negative S100β immunofluorescent staining in the intermediate zone (IZ). Wnt canonical loss-of-function during migration does not alter cell fate. Graph shows quantification of the percentage of SatB2 positive electroporated neurons in the IZ; n = 7 and 3 brains (control and dnTCF4, respectively), Kruskal-Wallis, P = 0.5238 for lower IZ and P > 0.9999 for upper IZ **(g)** Neurolucida reconstruction of control and dnTCF4 electroporated cells at P14. **(h)** Morphometric analysis of apical and basal dendrites revealed normal parameters in late arriving dnTCF4 electroporated cells; n = 6 brains, Mann-Whitney, from

left to right: $P = 0.3053$, $P = 0.5714$, $P = 0.5130$, $P = 0.4740$ and $P = 0.675$. **(i)** Validation of the shWnt3A construct by qRT-PCR on RNA from transfected HEK293T cells; n = 3 experiments, Mann-Whitney. **(j)** Analysis of cell distribution at P3; n = 10 and 8 brains (control, shWnt3a, respectively), 2way ANOVA. Graphs display mean±s.e.m. ns = non-significant, *: $P < 0.05$, **: $P < 0.01$, ***: $P < 0.001$. Bar = 20 µm (a, f and g) and 100 µm (b and d).

Supplementary Figure 2

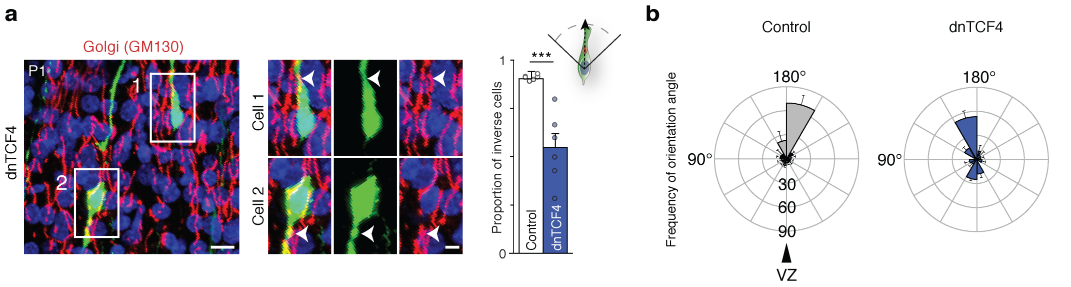

Wnt LOF during radial migration perturbs cellular orientation. (a) Gm130 immunostaining in red indicates the localization of the Golgi apparatus (arrowheads) at P1. The proportion of cells with the Golgi facing the pial surface in CP was quantified and graphed; n = 6 brains, Mann-Whitney. **(b)** Radial representation of the frequency of orientation angle distribution in migrating dnTCF4 over-expressing cells in CP at P1. Graphs display mean±s.e.m. ***: $P < 0.001$. Bars = 20 µm.

Supplementary Figure 3

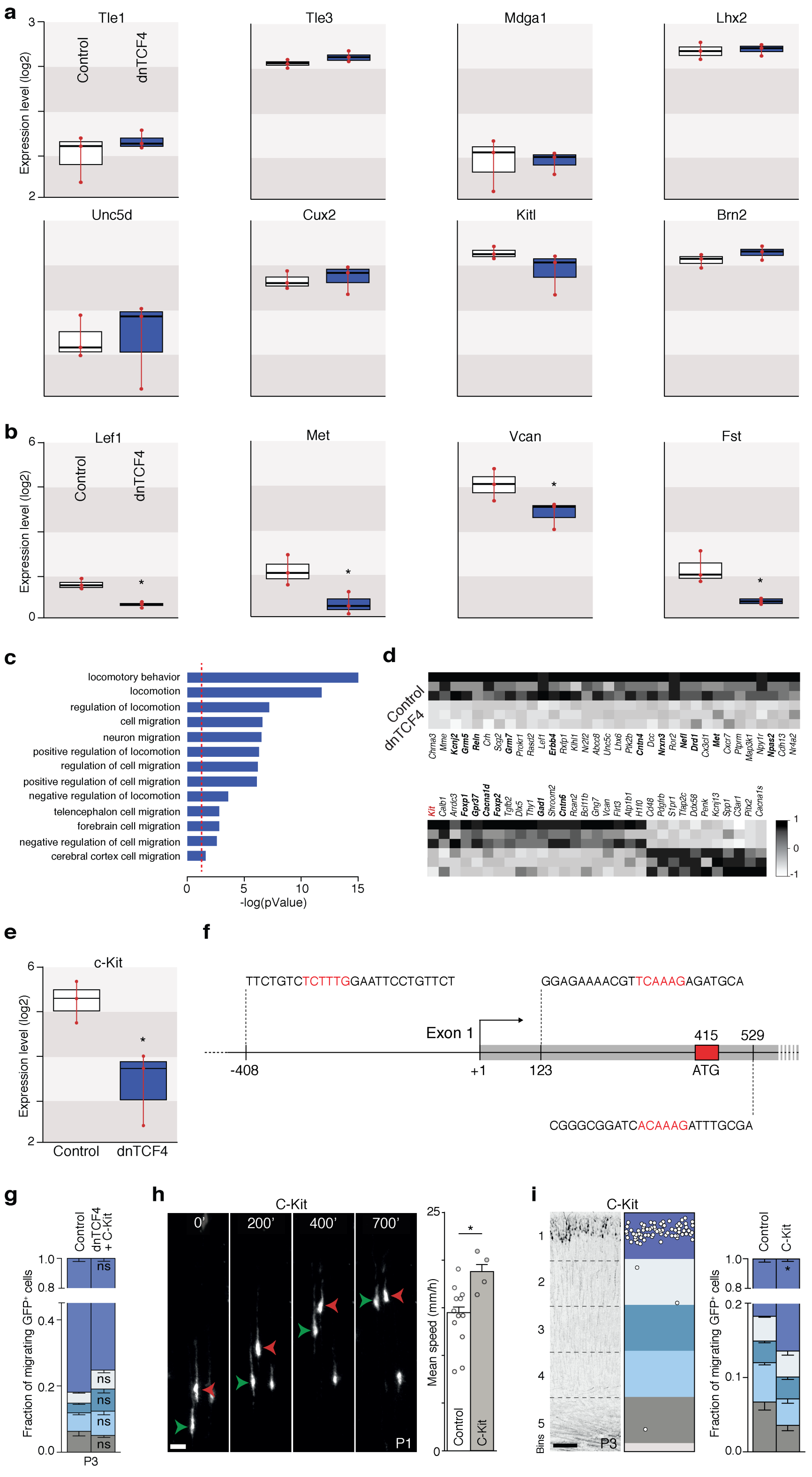

Transient Wnt LOF does not change cellular identity, but strongly affects migration-related gene expression. (a) Expression levels of 8 different characteristic markers of L2/3 pyramidal neurons in P0 migrating cells showing no differences between control and dnTCF4 groups; n = 3 experiments, box plots represent 25% and 75% percentiles and the median, Student's *t* test. **(b)** Expression levels of 4 known Wnt targets genes down regulated by dnTCF4 in our RNA sequencing data; n = 3 experiments, box plots represent 25% and 75% percentiles and the median, Student's *t* test. **(c)** Gene enrichment analysis shows significant enrichment of all migration related gene ontologies among DEGs. **(d)** Heatmap shows the relative expression of migration-related genes in control and dnTCF4 cells. C-Kit is highlighted in

red. **(e)** C-Kit expression level shows a robust decrease in dnTCF4 compared to control migrating cells; n = 3 experiments, box plots represent 25% and 75% percentiles and the median, Student's *t* test. **(f)** C-Kit promoter and exon 1 sequences contain three potential TCF/LEF binding sites. **(g)** Analysis of cortical cell distribution at P3; n = 9 and 12 brains (control and dnTCF4+C-Kit, respectively), 2way ANOVA. **(h)** Video time lapse images demonstrating that overexpression of C-Kit accelerates speed of locomotion; quantification based on n = 13 and 4 brains (Control and C-Kit, respectively), Mann-Whitney. **(i)** Fraction of migrating cells at P3 is shown in each bin after C-Kit overexpression; n = 10 and 9 (control and C-Kit, respectively), 2way ANOVA. Graphs display mean±s.e.m. ns = non-significant, *: P < 0.05. Bars = 20 µm (h) and 100 µm (i).

Supplementary Figure 4

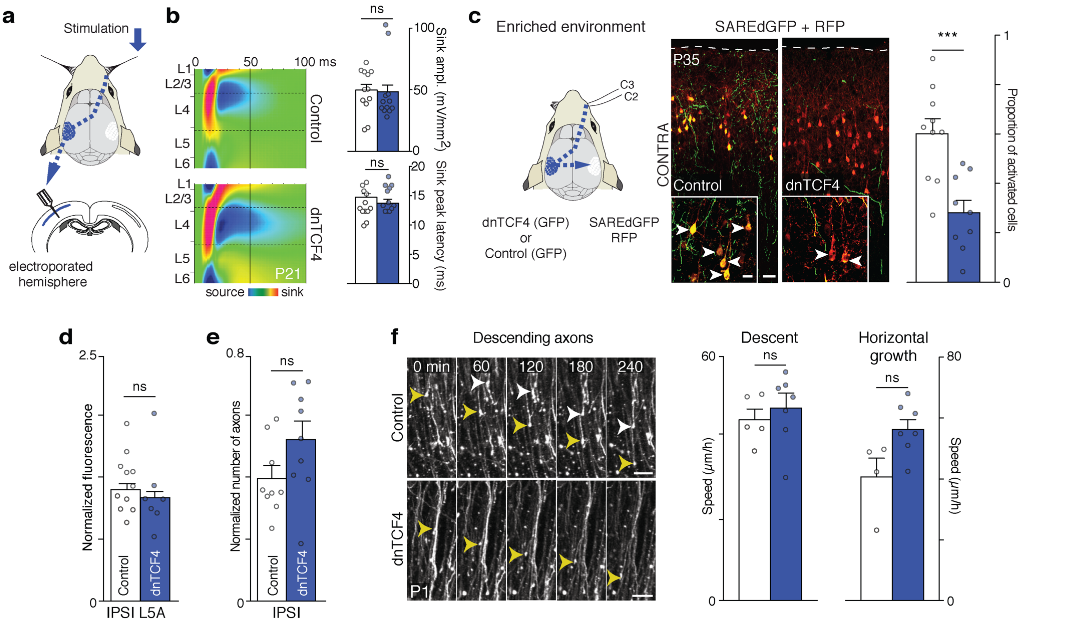

Late arriving CPNs display perturbed long-range cortical connections but their axonal growth pattern appears normal. (a) Schematic of intracortical recording of whisker-stimulation evoked potentials. **(b)** Averaged current source density map of the somatosensory barrel field when recording in hemispheres contralateral to whisker stimulation. Graphs illustrate the quantifications of the sink peak amplitude and latency; n = 12 animals, Wilcoxon test, P = 0.9697 for amplitude and P = 0.5445 for latency. **(c)** Schematic of the enriched environment experiment on P35 animals, following complete whisker-trimming on the dnTCF4 electroporated side and partial trimming on the opposite side. The activity-reporter (SAREdGFP) and RFP expressed in the hemisphere opposite to the dnTCF4 electroporation shows decreased GFP expression in homotypic L2/3 (arrowheads in magnified inserts point at activated cells) as quantified on the right (proportion of GFP/RFP positive cells); n = 9 and 10 brains (control and dnTCF4, respectively), Student's *t* test. **(d)** Average fluorescence of L5A ipsilateral normalized by the density of cells; n = 11 and 7 brains (control and dnTCF4, respectively), Mann-Whitney, P = 0.7859. **(e)** Number of vertically oriented axons in the electroporated hemisphere normalized by the density of electroporated cells at P14; n = 9 brains, Student's *t* test, P = 0.1234. **(f)** Time lapse of descending axons. Analysis of descent and horizontal growth shows similar progression speed of axons of control and dnTCF4 electroporated cells; n = 5 and 7 brains (control and dnTCF4, respectively), Student's *t* test, P = 0.3445 for descent and P = 0.1143. See also Supplementary Movie 5. Graphs display mean±s.e.m. ns = non-significant, ***: P < 0.001. Bars = 20 µm (c, insert), 50 µm (f) and 100 µm (c, overview).

Supplementary Figure 5

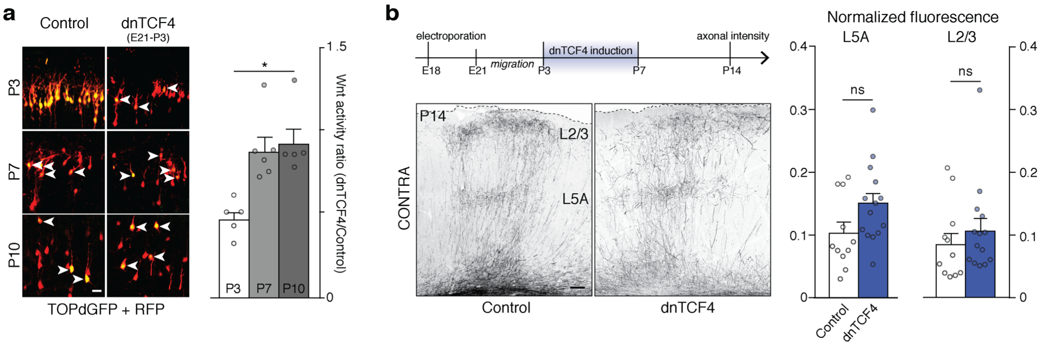

Transient Wnt LOF is not directly responsible for the deficient axonal development. (a) Canonical Wnt signaling activity reporter (TOPdGFP) and RFP co-expression at P3, P7 and P10 corresponding to 0, 4 and 7 days after the end of dnTCF4 induction, respectively; arrowheads point at TOPdGFP positive cells. Quantification of Wnt-activity ratio proves that 4 days without dnTCF4 induction are sufficient to restore normal Wnt-activity levels; n = 6 brains, Kruskal-Wallis. **(b)** Timeline of experiments showing postponed dnTCF4 induction (between P3-P7, i.e. post migratory period of axonal growth). Following postponed dnTCF4 induction contralateral somatosensory cortices show similar axonal arborization as controls as confirmed by the intensity measurements of the contralateral L5A and L2/3 regions normalized by ipsilateral L5A intensities; n = 11 and 14 brains (control and dnTCF4, respectively), Mann-Whitney, P = 0.0507 for L5A and P = 0.2915 for L2/3. Graphs display mean±s.e.m. ns = non-significant, *: P < 0.05. Bars = 50 µm (a) and 100 µm (b).

Supplementary Figure 6

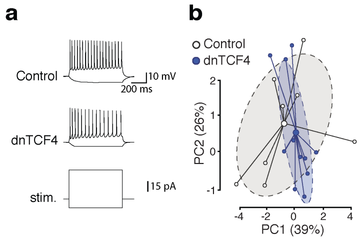

Transient Wnt LOF does not perturb intrinsic electrophysiological properties. (a) Sample traces showing subthreshold and supra-threshold responses during electrophysiological recordings in current-clamp mode of layer 2/3 neurons from acute slices of control and dnTCF4 electroporated animals. **(b)** The principal component (PC) analysis based on the electrophysiological parameters detailed in Table 1 shows large overlap between the control and dnTCF4 groups.

Supplementary Figure 7

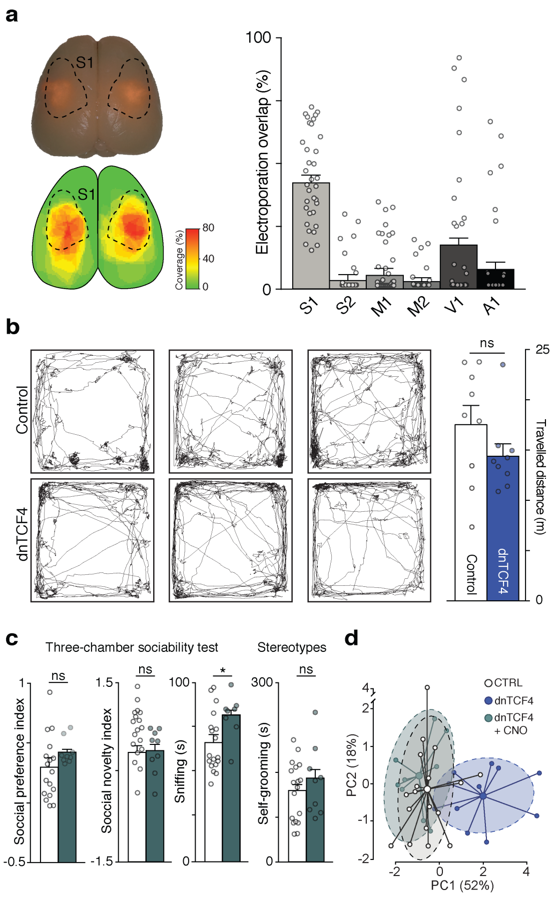

Impaired social and compulsive behaviors are rescued by post-natal chemogenetic activation of late-arrive neurons. (a) Extent of electroporated neurons with the main cortical areas: somarosensory (S), motor (M), visual (V) and auditory (A) cortex. **(b)** Six representative example track-lines reflecting spontaneous locomotor activity during the open field test and quantification of the total travelled distance; n = 9 animals, Mann-Whitney, P = 0.4534. **(c)** Sociability, interaction (sniffing), and tendency for repetitive movements (self-grooming) are rescued by enhancing neuronal activity; n = 18 and 9 animals (control and dnTCF4+CNO, respectively), Mann-Whitney, P = 0.1154 for social preference, P = 0.2304 for social novelty, P = 0.5467 for sniffing and P = 0.4689 for self-grooming. **(d)** PC analysis plots of autistic-like variables. Graph display mean±s.e.m. ns = non-significant, *: P < 0.05.
