# Supplementary material for: Perturbed Wnt signaling leads to neuronal migration delay, altered interhemispheric connections and impaired social behavior
Source: Nat Commun. 2017 Oct 27;8:1158. doi: 10.1038/s41467-017-01046-w (PMC5660087; doi:10.1038/s41467-017-01046-w)
Supplement: Supplementary file 3 — Description of Additional Supplementary Files [file 41467_2017_1046_MOESM3_ESM.pdf]

Description of Additional Supplementary Files

File Name: Supplementary Movie 1
Description: **Confocal time-lapse video of TOPdGFP/RFP electroporated L2/3 neurons migrating in the cortical plate (CP) at P0**. The intensity ratio of TOPdGFP/RFP is color-coded. Examples of two cells in the lower (low) and upper (high) CP show a dynamic and generally increasing cellular intensity ratio (Wnt-activity) during migration.

File Name: Supplementary Movie 2
Description: **Confocal time-lapse video illustrating control, dnTCF4 and dnTCF4 + C-Kit electroporated L2/3 neurons migrating in the CP at P1**. Analysis of radial migration revealed a reduced speed during locomotion in dnTCF4 electroporated cells. C-Kit co-expression restores the speed of migration. Green arrowhead marks the starting point, red arrowhead follows the cellular movement.

File Name: Supplementary Movie 3
Description: **Confocal time-lapse videos illustrating dynamics of the leading process length of control and dnTCF4 electroporated L2/3 neurons in the CP at P1**. Leading process length of dnTCF4 electroporated cells decreased over time compared to control cells. Orange arrowhead marks the tip of the leading process while the withe arrowhead point to the cell body.

File Name: Supplementary Movie 4
Description: **Confocal time-lapse videos illustrating leading process inversion of dnTCF4 electroporated L2/3 neurons in the CP at P1.** Electroporated dnTCF4 cells display an increased inversion of the leading process (arrowhead) compared to control cells.

File Name: Supplementary Movie 5
Description: **Confocal time-lapse videos illustrating axonal growth of control and dnTCF4 electroporated L2/3 neurons.** Speed analysis of descent and horizontal growth of axons shows similar progression in control and dnTCF4 electroporated brains. Green arrowhead marks the starting point, red arrowhead follows a growing axon.

File Name: Supplementary Data 1
Description: **RNA sequencing of migrating control and dnTCF4 electroporated neurons at P0.** The first sheet contains the raw and normalized read counts detected in all samples (n = 3), fold change (FC), counts per million (CPM), P-Value and false discovery rate of all expressed genes. The second sheet shows a selection of 207 genes that were considered differentially expressed between control and dnTCF4 samples. The third sheet contains a selection of 65 differentially expressed, migration-related genes. Genes related to both migration and autism are highlighted in yellow.
